# Estimating the role of casual contact from the community in transmission of *Bordetella pertussis *to young infants

**DOI:** 10.1186/1742-7622-4-15

**Published:** 2007-10-19

**Authors:** Aaron M Wendelboe, Michael G Hudgens, Charles Poole, Annelies Van Rie

**Affiliations:** 1 Department of Epidemiology, School of Public Health, University of North Carolina at Chapel Hill, Chapel Hill, NC, USA; 2 Department of Biostatistics, School of Public Health, University of North Carolina at Chapel Hill, Chapel Hill, NC, USA

## Abstract

The proportion of infant pertussis cases due to transmission from casual contact in the community has not been estimated since before the introduction of pertussis vaccines in the 1950s. This study aimed to estimate the proportion of pertussis transmission due to casual contact using demographic and clinical data from a study of 95 infant pertussis cases and their close contacts enrolled at 14 hospitals in France, Germany, Canada, and the U.S. between February 2003 and September 2004. A complete case analysis was conducted as well as multiple imputation (MI) to account for missing data for participants and close contacts who did not participate. By considering all possible close contacts, the MI analysis estimated 66% of source cases were close contacts, implying the minimum attributable proportion of infant cases due to transmission from casual contact with community members was 34% (95% CI = 24%, 44%). Estimates from the complete case analysis were comparable but less precise. Results were sensitive to changes in the operational definition of a source case, which broadened the range of MI point estimates of transmission from casual community contact to 20%–47%. We conclude that casual contact appears to be responsible for a substantial proportion of pertussis transmission to young infants.

**Medical subject headings (MeSH): **multiple imputation, pertussis, transmission, casual contact, sensitivity analysis, missing data, community.

## Introduction

Pertussis disease is poorly controlled among infants, adolescents, and adults in developed countries despite high immunization coverage rates [1-3] of ≥ 93 percent for both the primary infant series [4-6] and the booster at school entry [7]. *Bordetella pertussis *is reported to be among the most contagious pathogens in humans as an average of 15 secondary infections arise from a single case in a susceptible population [8]. Public health messages focus on the importance of transmission from close contacts [9-11] implying that pertussis transmission due to casual contact from community members is not appreciable.

Several studies investigated the disease dynamics of *B. pertussis*, especially as they relate to the transmission of the bacteria to young infants. This has been done by collecting diagnostic information on close contacts and assigning, where possible, one person as the most probable source of infection (where the difficulty in identifying the source case usually lies in identifying even one symptomatic source case as opposed to choosing from multiple potential cases). These studies identified close/household contacts as a source of infection for 40–53 percent of young infants with pertussis [12-16]. However, none of these studies investigated whether the remaining 47–60 percent of transmission was due to casual contact in the community or caused by transmission from unidentified close contacts as no attempt was made to rule out transmission from all identifiable household and other close contacts.

In order to infer transmission from a casual contact source, it is necessary to conclusively determine transmission did not occur from a close contact source. Several obstacles have hindered previous studies' ability to eliminate the possibility that transmission came from a close contact source. First, missing data due to non-participation of close contacts and participants' refusal to provide specimens for laboratory diagnostic testing has been high [12] or unreported, limiting the ability to determine whether a given contact would have been identified as the source of the index case's infection had the data not been missing. Second, diagnosing pertussis is often problematic since many adolescent and adult pertussis cases do not present with the typical symptoms of whooping cough [17-19]. This is further complicated by the lack of a highly sensitive and specific laboratory diagnostic method [10,20]. Third, inter-person variability in the incubation and infectious periods [21,22] may result in failure to identify source cases if their incubation or infectious periods lie in the tails of the distributions not captured by standard definitions [16,23-25]. Finally, it is uncertain whether individuals with asymptomatic infection can transmit pertussis [13,26,27]. In the absence of sound evidence for or against the infectiousness of asymptomatic infections, the systematic exclusion of asymptomatically infected individuals as possible source cases (as done in all previous studies) may bias the results.

In this paper we estimated the minimum proportion of infant pertussis cases due to transmission from casual contact sources using information from a study designed to identify the source of infection in young infants. Results from multiple diagnostic tests (including polymerase chain reaction [PCR], culture, and paired serology) were available on household contacts and non-household persons with close contact with the infant. To adjust for missing data arising from non-enrollment or failure to provide diagnostic specimens, multiple imputation (MI) analysis was used. MI is a widely accepted method to account for missing data and is superior to complete case analyses for two reasons.

First, as the amount of missing data increases, the results from complete case analyses suffer a greater loss in precision than results obtained by MI analyses [28,29]. Second, when data are not missing completely at random (MCAR) and the missing data mechanism is appropriately specified, MI will produce less biased estimates than a complete case analysis to the extent that missingness is associated with the observed data [28,30]. This is particularly germane to this pertussis study since missingness is likely dependent on relationship to the index case. In addition to MI, sensitivity analyses were conducted to determine the effect of varying the sensitivity and specificity of the source case definition, including an analysis allowing for transmission from individuals with asymptomatic infection.

## Analysis

### Methods

#### Study design, participants, and data collection

A prospective multi-center epidemiologic study was conducted from February 3, 2003 through September 15, 2004 in 14 hospitals in four countries: Canada, France, Germany, and the U.S. [31]. Approval from Institutional Review Boards was obtained at each participating site and written informed consent was obtained from each participant. Partially vaccinated and unvaccinated infants aged ≤ six months diagnosed with laboratory confirmed pertussis (by PCR or culture) were invited to participate. Upon enrollment of the infant index case, all household members and eligible non-household contacts were recruited. Non-household contacts were eligible if they were in contact with the infant during the month prior to symptom onset in the index case and were either: 1) a full-time caretaker (> 30 hours/week) of the infant index case or 2) had an acute cough illness lasting at least seven days in the month prior to the pertussis diagnosis in the index case.

Demographic and clinical information relevant to pertussis was collected on each index case. During the interview with the parent of the index case, information was collected on all household members and eligible non-household contacts, independent of their participation in the study. Information collected pertained to the contacts' relationship and amount of contact with the index case, and the presence and duration of symptoms of cold or cough in the contact during the month prior to enrollment of the index case. All contacts of the index case were also invited to be interviewed face-to-face using a standard questionnaire to obtain relevant demographic and clinical data.

All participants were asked to provide a sample collected via nasopharyngeal aspirate or swab for culture and PCR detection of *B. pertussis *and an acute blood sample for immunoglobulin-G (IgG) anti-pertussis toxin antibody (anti-PT) detection by enzyme-linked immunosorbent assay (ELISA). One month later, data on the presence of cough and cold-like symptoms and a convalescent blood sample were collected from participating contacts.

All PCR and serum samples were sent to the Reference Laboratory for Whooping Cough and other Bordetelloses Institut Pasteur in Paris. Nasopharyngeal specimens were analyzed with real-time PCR using the IS481 target and measurement of anti-PT IgG, using ELISA, were performed according to recent recommendations[32].

#### Case definitions

Source cases were either laboratory confirmed or epidemiologically linked pertussis cases with onset of cough two to 48 days prior to symptom onset in the infant index case [9,22]. When two or more contacts reported symptoms prior to the index case, the person with the earliest symptom onset (within the defined time-frame) was assigned as the source case. Multiple source cases were allowed if their symptom onset was reported to be the same day. Laboratory confirmation was defined by at least one of the following: positive culture, positive PCR, a ≥ four-fold change [33] in IgG titer to anti-PT between acute and convalescent serum samples, or a single anti-PT IgG antibody titer ≥ 125 EU/mL [34,35]. For immunized children aged three months to three years and four to seven years, anti-PT titer results were not used for confirmation of pertussis as they may be influenced by recent vaccination. Epidemiologically linked cases were defined as persons in contact with the infant index case in the month preceding symptom onset in the index case, who had an acute cough illness lasting ≥ two weeks, but no laboratory confirmation of pertussis [36]. Laboratory confirmed asymptomatic cases of pertussis were also identified. They met the same criteria as laboratory confirmed symptomatic cases, but did not report any cough or cold symptoms.

#### Primary outcome analysis

The proportions of infant pertussis cases infected by close contacts and casual contacts were estimated using complete case and multiple imputation analyses. 95 percent confidence intervals were calculated in Stata 8.2 (College Station, TX) for the complete case analysis and in Excel 2002 for the MI analysis [37]. Generalized estimating equations (GEE) regression using an exchangeable working correlation matrix was used for the MI models to allow possible clustering within households. GEE regression models were fit using SAS 9.3.1 (Cary, NC).

The complete case analysis included all index cases for whom (1) complete enrollment of all eligible close contacts (household and non-household) was achieved and (2) all enrolled contacts had complete diagnostic data, defined as information on symptoms, results from PCR, and results from at least one serum sample.

GEE regression models were fit to build predictive models for MI. As the demographic and symptom history data (including age, relationship to the index case, amount of contact with the index case, household size, presence of cold/cough symptoms, presence of cough lasting at least two weeks, and continent of birth) were available on all identified close contacts, independent of study participation, missing data from non-participants were treated the same as missing data from participants. This information was used as predictor variables in building the imputation model. The imputation model predicted the probability that each symptomatic contact with missing data was the source case for the index case. We created 10 imputed data sets, adapting the methods described by Raghunathan et al. [30] for non-sequential logistic regression. Indicator variables incorporating interactions between three explanatory variables (relationship to the index case, severity of symptoms, and amount of contact with the infant) were generated. For example, one variable indicated all parents with a cough lasting ≥ two weeks and contact with the index case for > five hours/day; another indicated all siblings with a cough lasting ≥ two weeks and contact with the index case for > five hours/day. Household size (a count variable) was also included in the predictive model. The average number of source cases from the 10 imputed data sets was used as the estimated number of source cases. Approximate 95% CIs were calculated using a standard normal approximation and the multiple imputation variance estimate [28,30].

The missing data mechanism was assumed to be ignorable. An ignorable missing data mechanism in this study means that missingness (*i.e*., inability to make a pertussis diagnosis due to non-enrollment or failure to provide diagnostic specimens) was independent of being a source case, conditional on the observed data (*i.e*., age, relationship to the index case, amount of contact with the index case, household size, presence of cold/cough symptoms, and presence of cough lasting at least two weeks).

#### Sensitivity analyses

Sensitivity analyses were conducted on the multiply imputed data to assess the impact of the sensitivity and specificity of the source case definition. In the *specific source case definition *analysis, specificity was increased by requiring symptom onset in the source case to occur seven to 30 days prior to symptom onset in the index case [23-25,38,39]. In the *sensitive source case definition *analysis, sensitivity was increased by allowing individuals with laboratory confirmed asymptomatic pertussis to be source cases when no other source case could be identified for an infant index case. In the latter analysis, the outcome value (source case status) was imputed using MI for all close contacts with missing data, independent of symptoms.

#### Analysis of infant characteristics associated with transmission from a casual contact source

Risk ratios (RR) and 95 percent confidence intervals (CI) were calculated to identify risk factors for pertussis in infants as a result of *B. pertussis *transmission by casual community contact. Potential risk factors included age, sex, time from symptom onset to diagnosis, vaccination status, hospitalization status, household size, location of child care, presence of an adolescent close contact, enrollment of at least one non-household close contact, and continent of residence. Data from each imputed data set were used to fit modified Poisson regression models (which uses a robust error variance) to estimate the log binomial model [40]. (Modified Poisson regression was used to overcome non-convergence problems using binomial regression.) Crude RR estimates were computed by averaging the corresponding estimates from the 10 fitted models with each potential risk factor as the lone covariate. Fully adjusted RR estimates were computed similarly based on models including each of the above characteristics of the infant index cases. Approximate 95% CIs were calculated using standard normal approximations and multiple imputation variance estimates. The fully adjusted model included each of the above characteristics of the infant index case as a means of conducting an exploratory analysis.

## Results

### Participants

The study population comprised 95 infant index cases and 460 identified eligible close contacts. A pair of twins were enrolled with identical onset dates and contact patterns and thus treated as a single case, leaving a total of 94 cases. Fifty-six contacts refused enrollment. Complete enrollment (*i.e*., enrollment of all household and eligible non-household close contacts) was achieved for 70 index cases and their 316 contacts. Outcome (case) status was missing for 51 (12.6%) of the 404 participating contacts: 21 symptomatic and 30 asymptomatic participating contacts (Table 1). Complete information (*i.e*., complete enrollment and complete laboratory data) was available for 45 index cases and their 193 contacts.

**Table 1:** Distribution of all identified close contacts to infant index cases, stratified by enrollment status, information on outcome (case) status, presence of symptoms, and household contact type.

	**Adult Household**		**Child Household**		**Non-household**		**Total**	
	**n**	**%**	**n**	**%**	**n**	**%**	**n**	**%**
Enrolled, known outcome (case) status	199	84.3	96	63.6	58	79.5	353	76.7
Enrolled, missing outcome status	7	3.0	31	20.5	13	17.8	51	11.1
Symptomatic	1	0.4	14	9.3	6	8.2	21	4.6
Asymptomatic*	6	2.6	17	11.2	7	9.6	30	6.5
Not enrolled	30	12.7	24	15.9	2	2.7	56	12.2
Symptomatic	11	4.7	12	7.9	2	2.7	25	5.4
Asymptomatic	19	8.1	12	7.9	0	0.0	31	6.7
**Identified contacts**	**236**	**100.0**	**151**	**100.0**	**73**	**100.0**	**460**	**100.0**

*Multiple imputation was not used to impute source case status among asymptomatic persons in the main analysis (and the specific case definition analysis), because they are, by definition, not a source case in these analysis.

The distribution of all identified close contacts to the infant index cases, stratified by household contact type (adult household, child household and non-household status) and enrollment status is shown in Table 1. Among study participants, child household contacts and non-household contacts were more likely than adult household contacts to be missing outcome status: odds ratio (OR) = 9.2 (95 percent CI = 3.9, 21.6) and OR= 6.4 (95 percent CI = 2.4, 16.7), respectively. Age (OR = 1.0, 95 percent CI = 0.98, 1.01), symptoms before the first visit (OR = 0.7, 95 percent CI = 0.4, 1.2), and level of contact with the infant (OR = 1.0, 95 percent CI = 1.0, 1.1) were not associated with missing outcome status. However, relationship with the infant index case was associated with non-participation as siblings (OR = 2.5, 95 percent CI = 1.1, 5.4) and "other" contacts (OR = 3.3, 95 percent CI = 1.6, 7.0) were less likely than parents to enroll. Thus, the data were not MCAR among both participants and non-participants.

### Complete case and multiple imputation analyses

A source case could not be identified among the close contacts for approximately one in three infants with pertussis. Even though the MI analysis identified on average eight additional source cases, the estimates on the proportion of infants for whom no source case could be identified were similar between the MI (34 percent, 95 percent CI = 24 percent, 44 percent) and complete case analyses (31 percent, 95 percent CI = 17 percent, 45 percent), with the estimate from the MI analysis being slightly more precise (Table 2). Assuming missingness among participants was missing at random and no misclassification of outcome status occurred, it can be inferred that infants for whom no close contact source case was identified were infected through casual contact with an infectious case of pertussis in the community. Multiple source cases were identified for four index cases.

**Table 2:** Proportion of infants for whom a close contact source case was identified versus for whom a community contact source was inferred stratified by analysis type.

Analysis	Infants included in analysis		For whom a source case was identified			For whom contact in community likely responsible		
	**n**	**%**	**n**	**%**	**95%CI**	**n**	**%**	**95%CI**
Complete Case	45	47.9	31	68.9	(55.4, 82.4)	14	31.1	(17.6, 44.6)
Primary Multiple Imputation	94	100.0	62	65.7	(55.7, 75.8)	32	34.2	(24.2, 44.3)
Specific Source Case Definition*	94	100.0	50	53.4	(42.3, 64.4)	44	46.6	(35.6, 57.6)
Sensitive Source Case Definition**	94	100.0	75	79.7	(69.8, 89.6)	19	20.3	(10.4, 30.2)

*The specific source case definition analysis requires symptom onset in primary cases to occur 7–30 days prior to symptom onset in the index case

**The sensitive source case definition allows asymptomatically infected persons to be primary cases in the absence of identifying a symptomatic primary case

### Sensitivity analyses

In the *specific source case definition *analysis, using the more restrictive period of incubation and infectiousness (seven to 30 days instead of two to 48 days), a source case was identified using MI for an average of 50 (53 percent) index cases, implying that casual contact in the community was solely responsible for 47 percent (95 percent CI = 36 percent, 58 percent) of transmission to young infants (Table 2). In the *sensitive source case definition *analysis, allowing laboratory confirmed asymptomatically infected persons to be source cases, a source case was identified using MI for an average of 75 (80 percent) infants, implying the remaining 20 percent (95 percent CI = 10 percent, 30 percent) of index cases were infected by casual contact in the community (Table 2).

### Predictors of transmission from the community

Risk ratios (95 percent confidence intervals) for characteristics of the index cases associated with an unidentified source of transmission, implying transmission from casual contact in the community, are presented in Table 3. Time from symptom onset to diagnosis, childcare outside the home, and household size were factors we *a priori *thought might be associated with community transmission. No strong associations were identified between infant characteristics and having a casual contact as the source case, as most of the point estimates were near the null value and bounded by relatively wide confidence intervals. The strongest association observed was infants born in North America had a greater risk of having a source case being a casual contact than infants born in Europe (RR = 3.29; 95 percent CI = 0.86, 12.7). It is interesting to note the reversal of the estimated effect of being partially vaccinated in the crude and fully adjusted models. No single characteristic was identified as a confounder for this observation.

**Table 3:** Characteristics of index cases analyzed to determine associations with having a casual contact source case.

	**Crude**		**Fully Adjusted**	
**Characteristic of infant index case**	**RR**	**95%CI**	**RR**	**95%CI**
Time to diagnosis				
< 13 days	1.00	(...)	1.00	(...)
14–20 days	1.53	(0.64, 3.7)	1.22	(0.45, 3.3)
21–27 days	0.59	(0.13, 2.7)	0.49	(0.10, 2.5)
28+ days	1.92	(0.65, 5.7)	1.60	(0.40, 6.4)
Age				
< 2 months	0.75	(0.28, 2.0)	0.52	(0.08, 3.3)
2–3 months	0.98	(0.39, 2.5)	1.33	(0.43, 4.2)
4–6 months	1.00	(...)	1.00	(...)
Male gender	1.10	(0.52, 2.3)	1.20	(0.55, 2.6)
Partially vaccinated	1.57	(0.74, 3.3)	0.59	(0.13, 2.7)
Household size	1.03	(0.87, 1.2)	1.07	(0.85, 1.3)
Non-household contact enrolled	0.93	(0.42, 2.0)	1.04	(0.41, 2.6)
Childcare outside the home	1.57	(0.48, 5.2)	1.68	(0.39, 7.2)
Adolescent in close contact	0.70	(0.31, 1.6)	0.45	(0.14, 1.4)
Hospitalized	0.55	(0.25, 1.1)	0.87	(0.30, 2.5)
Continent of residence				
Europe	1.00	(...)	1.00	(...)
North America	2.59	(0.91, 7.4)	3.29	(0.86, 12.7)

## Conclusion

In this study, we estimated the proportion of pertussis transmission to young infants due to casual contact with community members. We estimate that approximately one in three infants with pertussis is infected though casual contact with a source case in the community.

By collecting some data on all non-enrolled eligible close contacts (during the first interview with the parents of the infant index case) we employed an imputation model which accounted for missing data and thus utilized observed data from all 460 identified close contacts of the 94 index cases. Assuming that data were missing at random and that there was no misclassification of the outcome status when using PCR and serological diagnostic methods, we inferred that in the absence of identifying a source case among close contacts of the index infant cases, pertussis transmission to these young infants was due to casual contact in the community.

The only other study to estimate the proportion of casual or community transmission was performed in the pre-vaccine era. In 1913–1914, Luttinger followed 2,310 pertussis cases who attended a whooping cough clinic [41]. The sources of infection, as reported by the guardian, were identified close contacts such as neighbor (56.8 percent), relative or friend (17.6 percent), school or nursery (9.6 percent), causal contact at movies, recreation pier, roof gardens, ferry, street, and public transportation (4.6 percent); and unknown (11.4 percent) [41]. Considering the dramatic change that widespread vaccination wrought on the epidemiology and disease dynamics of pertussis [42], the comparison of results is somewhat trivial. One explanation for the observed increase in the proportion of transmission due to casual contact in the vaccine era is that widespread vaccination reduces the severity of symptoms, allowing those infected with *B. pertussis *to continue their daily activities, thus shifting a greater proportion of transmission from the home to the community. It is also possible some close non-household contacts were not identified in this study as a result of asymptomatic and atypical, mild presentation of pertussis in vaccinated individuals [43].

When analyzing index case characteristics that could potentially be associated with the source of transmission, we failed to identify factors significantly associated with transmission due to casual contact from the community. Similar to our results, a study in 1988 attempted to identify risk factors for community-acquired pertussis, and found that exposure outside the household was the only significant predictor [44].

Several limitations to the study are important to note. First, we were unable to prove that the missing data mechanism was ignorable (i.e. was not dependent on source case status). Second, the identification of individuals with asymptomatic infections as the source case is limited by the unknown chronology of infection as those with asymptomatic infection could either have infected the index case or have been infected by the index case. Furthermore, transmission from asymptomatic infected individuals has not been conclusively demonstrated. In the context of infant pertussis, transmission may not require aerosol generation through coughing or sneezing as respiratory secretions are likely to be shared through activities such as kissing, cuddling, and singing. The finding that, in the sensitive source case definition sensitivity analysis, a source case was identified among close contacts for an additional 14% of infants underscores the potential role of these carriers in the transmission of *B. pertussis *to young infants.

In conclusion, this study presents evidence that casual contact from the community is responsible for 34 percent of pertussis transmission to young infants (20–47 percent including sensitivity analyses, and 10–60 percent including confidence intervals from sensitivity analyses). This proportion is higher than the 5–16 percent reported in the pre-vaccine era and provides insight in the transmission dynamics of *B. pertussis *beyond observations from prior vaccine era studies where no source case could be identified for 47–60 percent of infant index cases [12-16]. Our results indicate that in future efforts to control pertussis in infants, transmission from both close and casual community contacts may need to be addressed. The cocoon vaccination strategy [45] and the routine adolescent [1] and adult vaccination strategies may thus be complementary strategies in the control of infant pertussis.

## Abbreviations

*Bordetella pertussis (B. pertussis)*, confidence interval (CI), enzyme-linked immunosorbent assay (ELISA), generalized estimating equations (GEE), immunoglobulin (Ig), missing completely at random (MCAR), multiple imputation (MI), pertussis toxin (PT), polymerase chain reaction (PCR), odds ratio (OR), risk ratio (RR), Tetanus, Diphtheria and acellular Pertussis vaccine (Tdap).

## Competing interests

The author(s) declare that they have no competing interests.

## Authors' contributions

AMW managed the study, contributed to study conception and design, analyzed the data, and drafted the manuscript. MGH contributed to data analysis and critically reviewed the manuscript for intellectual content. CLP and AVR contributed to study design, interpretation of the results, and critically reviewed the manuscript for intellectual content. All authors read and approved the final manuscript.
